# Characterization of aspartyl aminopeptidase from *Toxoplasma gondii*

**DOI:** 10.1038/srep34448

**Published:** 2016-09-28

**Authors:** Jun Zheng, Ziying Cheng, Honglin Jia, Yonghui Zheng

**Affiliations:** 1 Harbin Veterinary Research Institute, CAAS-Michigan State University Joint Laboratory of Innate Immunity, State Key Laboratory of Veterinary Biotechnology, Chinese Academy of Agricultural Sciences, Maduan Street 427, Nangang District, Harbin 150001, P. R. China

## Abstract

Aminopeptidases have emerged as new promising drug targets for the development of novel anti-parasitic drugs. An aspartyl aminopeptidase-like gene has been identified in the *Toxoplasma gondii* genome (*TgAAP*), although its function remains unknown. In this study, we characterized *TgAAP* and performed functional analysis of the gene product. Firstly, we expressed a functional recombinant *Tg*AAP (r*Tg*AAP) protein in *Escherichia coli*, and found that it required metal ions for activity and showed a substrate preference for N-terminal acidic amino acids Glu and Asp. Then, we evaluated the function and drug target potential of *Tg*AAP using the CRISPR/Cas9 knockout system. Western blotting demonstrated the deletion of *Tg*AAP in the knockout strain. Indirect immunofluorescence analysis showed that *Tg*AAP was localized in the cytoplasm of the wild-type parasite, but was not expressed in the knockout strain. Phenotype analysis revealed that *TgAAP* knockout inhibited the attachment/invasion, replication, and substrate-specific activity in *T. gondii*. Finally, the activity of drug CID 23724194, previously described as targeting *Plasmodium* and malarial parasite AAP, was tested against r*Tg*AAP and the parasite. Overall, *TgAAP* knockout affected the growth of *T. gondii* but did not completely abolish parasite replication and growth. Therefore, *Tg*AAP may comprise a useful adjunct drug target of *T. gondii*.

Protozoan parasites from the phylum Apicomplexa are a global health threat because of their ability to infect humans and animals. *Toxoplasma gondii*, which causes toxoplasmosis, infects a wide range of warm-blooded animals, including humans. There are no clear symptoms of acute toxoplasmosis in healthy adults^1^,^2^. However, immunocompromised people, such as those infected with HIV, immunocompromised individuals receiving certain types of chemotherapy, or those who have recently received an organ transplant, may develop severe toxoplasmosis. Severe toxoplasmosis can cause encephalitis in the brain or retinochoroiditis in the eyes^3^. Latent toxoplasmosis produces no readily observable symptoms in most immunocompetent people^4^, during which tissue cysts can form in the retinas, lungs, heart, skeletal muscle, and brain. Cysts form in the central nervous system upon infection with *T. gondii* and persist for the lifetime of the host^5^. Recent studies have suggested that infection of toxoplasma causes behavioral changes^6^. It was estimated that 30–50% of the global population has been exposed to and may be chronically infected with latent toxoplasmosis according to serological studies^7^,^8^. The currently available therapeutic drugs for toxoplasmosis treatment elicit hypersensitivity syndrome and/or are toxic^9^,^10^. Also, no vaccine is currently available that would block the transmission of this parasite. Thus, identifying drugs with new modes and/or targets of action is essential for creating safer and more effective drugs that are urgently needed to treat toxoplasmosis.

Peptidase-catalyzed protein degradation is one of the most important processes providing amino acids for an organism^11^. Aminopeptidases catalyze the cleavage of N-terminal amino acids in proteins and peptides. The *T. gondii* genome appears to encode at least ten aminopeptidases: one leucyl aminopeptidase (LAP), one aspartyl aminopeptidase, one prolyl aminopeptidase, three aminopeptidases N, and four methionyl aminopeptidases. AAP belongs to the M18 family of peptidases that has been proposed as a novel antimalarial drug target^12^. The M18 family is widely distributed in bacteria and eukaryotes, and consists of metalloaminopeptidases that use metal ions to increase their substrate specificity for N-terminal acidic amino acids, beyond aspartic and glutamic acids. Until now, only a few M18 family peptidases have been characterized in detail, including *Saccharomyces cerevisiae* aminopeptidase I (MEROPS: M18.001)^13^, mammalian AAP (MEROPS: M18.002)^14^,^15^,^16^, and *Plasmodium falciparum* AAP (*Pf*M18AAP) (MEROPS: M18.003)^12^. Kinetic measurements and structure determination of AAP have been performed in *Pseudomonas aeruginosa*^17^. An aminopeptidase from the M18 peptidase family is predicted to be an AAP (ToxoDB: TGGT1_297970) in *T. gondii*, although its structure is unknown and its characterization has yet to be performed.

Important functional characterization of AAPs has been performed in parasites. AAP gene disruption/truncation experiments indicated that the enzyme was dispensable for malarial blood-stage replication but its production had an associated fitness cost for the parasite^18^. The protease knockdown resulted in parasite growth abnormalities, vascularization, and cell damage^12^. In this study, we report for the first time the cellular distribution, enzyme kinetics, and inhibition of *Tg*AAP. We constructed a new *T. gondii TgAAP* deletion strain to characterize the enzyme. Our results showed that *Tg*AAP is synthesized in the parasite cytosol. *TgAAP* deletion resulted in inhibition, but not abolition, of invasion and growth of *T. gondii* in cell culture, thus indicating that the enzyme may be a promising adjunct target for anti-toxoplasma drug development.

## Results

### Analysis of TgAAP amino acid sequence

**Figure 1:**
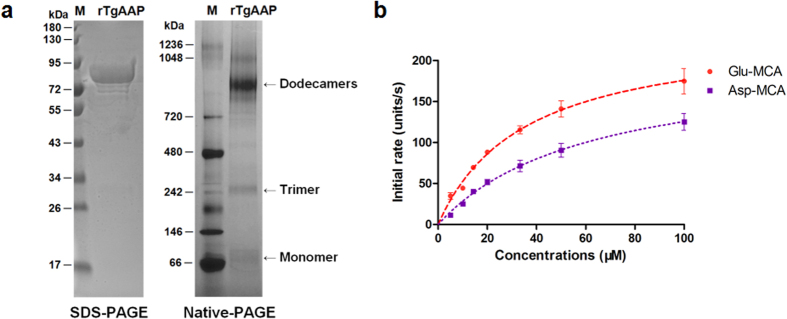
Characterization of the *TgAAP* protein product. (**a**) SDS-PAGE and native PAGE analysis of r*Tg*AAP. Left: SDS-PAGE (10% gel). Lane 1 (M), pre-stained protein standard (10–180 kDa). Lane 2, r*Tg*AAP [the protein was heated in 4× protein loading buffer (Solarbio, Beijing) at 100 °C for 5 min before loading]. Right: native PAGE (3–12% gel). Lane 1 (M), unstained protein standard (20–1200 kDa). Lane 2, r*Tg*AAP. (**b**) Enzyme activity of purified r*Tg*AAP. An initial assay with different concentrations of fluorogenic peptide substrates H-Glu-MCA and H-Asp-MCA is shown. One activity unit is defined as MCA (pM) released per mg of recombinant protein.

To investigate the similarity between *Tg*AAP and AAPs from other species, we cloned and sequenced the gene encoding *Tg*AAP from the *T. gondii* RH strain. The *TgAAP* coding sequence was 1521 bp long, consistent with the predicted sequence (ToxoDB accession number TGGT1_297970). Amino acid sequence alignment was performed with predicted AAPs from apicomplexan parasites, including *T. gondii*, *Eimeria tenella*, *Cryptosporidium muris*, *P. falciparum*, and *Thelleria annulata* (Fig. 1Sa). The alignment revealed that the predicted AAP functional domains (gray), especially Zn-binding sites (blue; residues 109, 300, 332, 377, and 471) and substrate-binding/catalytic sites (red; residues 111 and 333), may be conserved in apicomplexan parasites. A constructed phylogenetic tree revealed that *Tg*AAP was closely related to the predicted *E. tenella* AAP (51% identity; Fig. 1Sb). M18 aminopeptidases are reported to be dodecamers that form a tetrahedron^19^.

### Purification and multimeric nature of r*Tg*AAP

To explore the function and activity, we first expressed r*Tg*AAP protein in *E. coli.* The protein tagged with glutathione S-transferase (GST) was purified by affinity chromatography. The molecular weight of the protein was estimated by polyacrylamide gel electrophoresis (PAGE). This revealed a ~80 kDa band on SDS-PAGE, consistent with the predicted size (~56 kDa *Tg*AAP fused with the ~27 kDa GST tag; Fig. 1a). To investigate the multimeric state of the protein, r*Tg*AAP was analyzed using native PAGE. Native PAGE revealed a minor band (about 80 kDa) as well as two major oligomeric bands (about 960 kDa and 240 kDa). These sizes might correspond to r*Tg*AAP dodecamers and trimers.

### Enzyme activity of r*Tg*AAP

Next, r*Tg*AAP enzyme activity was analyzed against various synthesized peptidase substrates. The protein concentration (0.3 μM) at which the reaction rate was linear (25.72 μg/ml) was chosen for the assay. Data collected for the specific substrates H-Glu/Asp-MCA (7-methoxycoumarin-4-acetic acid) conformed to the Michaelis-Menten kinetics (Fig. 1b). The initial reaction rate of r*Tg*AAP with H-Glu-MCA was higher compared with the specific substrate H-Asp-MCA (Student’s t-test, *P* < 0.01, n = 3). Metal ion sensitivity was then investigated by assaying r*Tg*AAP activity after 30 min pre-incubation at 37 °C in Tris-HCl (50 mM, pH 7.5) containing bivalent metal chlorides (Sigma-Aldrich, St. Louis, USA). The enzyme was markedly activated by the addition of the bivalent metal cations Co^2+^, Mn^2+^, Fe^2+^, Ni^2+^, Mg^2+^, Ca^2+^, Cu^2+^, and Zn^2+^ (Table 1). Particularly with Co^2+^ (377.76 ± 56.36 U/s, initial rate ± STD), the initial rate increased more than 6-fold compared with the absence of metal ions (59.39 ± 2.72 U/s; Student’s t-test, *P* < 0.01, n = 3), followed by Mn^2+^ (151.3 ± 26.44 U/s), which enhanced enzymatic activity more than 2-fold (Student’s t-test, *P* < 0.01, n = 3). These results indicated that Co^2+^ was the most effective metal ion cofactor of r*Tg*AAP.

### Substrate specificity of r*Tg*AAP

r*Tg*AAP enzyme kinetics with fluorogenic synthetic substrates confirmed its classification as a member of the M18 AAP family. Acidic amino acids Glu and Asp were efficiently cleaved by r*Tg*AAP from the N-termini of synthetic substrates. Compared with H-Asp-MCA (*k*_cat_/*K*_m_ = 85.66 mM^−1 ^s^−1^), the catalytic efficiency of r*Tg*AAP was higher with H-Glu-MCA (*k*_cat_/*K*_m_ = 190.16 mM^−1 ^s^−1^; Student’s t-test, *P* < 0.01, n = 3) (Table 2). With Asn-MCA, r*Tg*AAP catalytic efficiency was even lower (*k*_cat_/*K*_m_ = 19.86 mM^−1 ^s^−1^; Student’s t-test, *P* < 0.01, n = 3). r*Tg*AAP had no preference or activity toward other synthesized substrates, such as His-, Ser-, and Pro-MCA, with enzyme efficiency values (*k*_cat_/*K*_m_) 2.35, 1.33, and 0.28 mM^−1 ^s^−1^, respectively.

### Generation of *TgAAP*-knockout and complemented parasites

**Figure 2:**
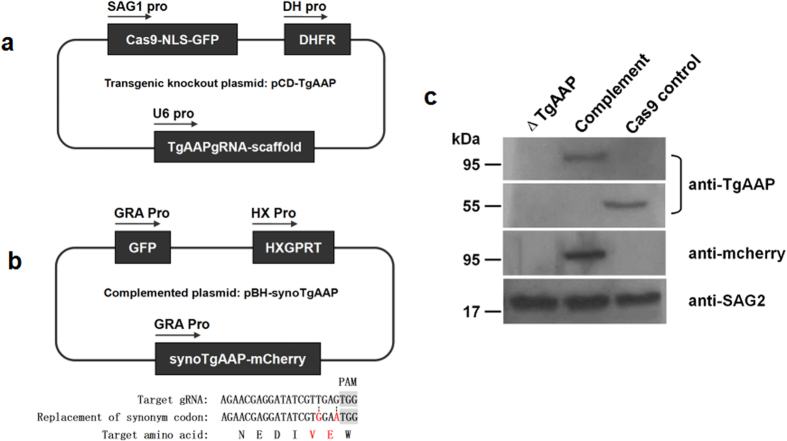
Cellular localization of *Tg*AAP. (**a**) Indirect immunofluorescence detection of *Tg*AAP in *T. gondii* RH strain. *Tg*AAP was detected with anti-r*Tg*AAP polyclonal antibodies (green). Host cell nuclei were visualized with DAPI (blue).

To investigate the function of AAPs in *T. gondii*, we proceeded to generate a *TgAAP*-knockout mutant (Δ*TgAAP*). A *TgAAP* gene-targeting plasmid, designated pCD-*Tg*AAP (Fig. 2a), was constructed using specific gRNA with a dihydrofolate reductase (*DHFR)* selectable marker cassette. Plasmid pBluescript II containing codons synonymous to *TgAAP* fused with *mCherry* and a hypoxanthine xanthine guanine phosphoribosyl transferase (*HXGPRT*) expression cassette derived from pHXNTPHA plasmid, designated pBH, were used to construct a *TgAAP*-complementing plasmid pBH-syno*Tg*AAP (Fig. 2b). After electroporation and selection, Δ*TgAAP*, complemented, and Cas9 control strains were verified by western blot analysis (Fig. 2c). Protein band ~56 kDa was observed in the lysate of the Cas9 control cell line, but not in the Δ*TgAAP* strain. The size corresponded to the predicted size (55897 Da) in ToxoDB. An ~95 kDa (*Tg*AAP fused with the mcherry tag) band was observed after probing the complemented strain lysate with anti-*Tg*AAP serum. This was confirmed using anti-mCherry monoclonal antibody. We also performed a PCR assay (Fig. 2S) to verify *TgAAP* knockout. All these experiments confirmed the absence of *TgAAP* in the Δ*TgAAP* strain.

### Cellular localization of *Tg*AAP

**Figure 3:**
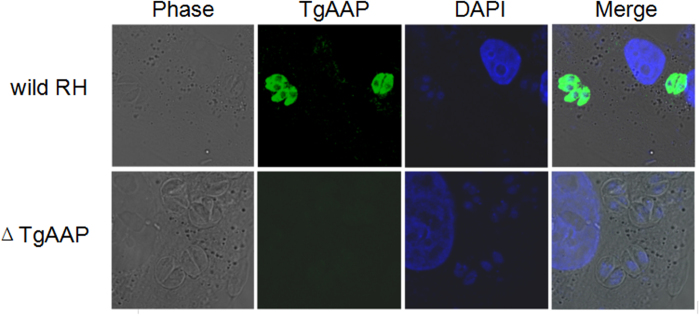
Knocking out *TgAAP.* (**a**) Schematic of the knockout plasmid. Knockout plasmid pCD-*Tg*AAP was constructed by inserting *DHFR* coding sequence and *TgAAP*-specific single guide RNA (gRNA) into plasmid pSAG1::CAS9-U6::sgUPRT (Addgene, #54467). The plasmid contains *Cas9*, nuclear localization signal (NLS), and expressed GFP fusion element. (**b**) Schematic of the complementing plasmid. To avoid recognition by Cas9, synonymous codon substitutions (not affecting *Tg*AAP amino acid sequence) were introduced in the *TgAAP* target located near a protospacer adjacent motif (PAM) in the complementing plasmid pGH-syno*Tg*AAP. mCherry tag was used to identify the complementing *Tg*AAP. *HXGPRT* expression element was used to screen for complemented parasite lines. (**c**) Western blotting analysis of *TgAAP*-knockout (Δ*TgAAP*) and complemented parasites. *Tg*AAP was detected with a mouse anti-*Tg*AAP serum in Cas9 control and complemented strains but not in Δ*TgAAP*. The enzyme was also detected with a mouse monoclonal anti-mCherry antibody in the complemented strain. *Tg*SAG2, detected using mouse anti-*Tg*SAG2 serum, was used as a loading control. Primary antibodies were targeted by horseradish peroxidase-labeled goat anti-mouse secondary antibodies (Sigma, St Louis, USA). Pre-stained protein marker (10–180 kDa) is shown.

Confocal laser scanning microscopy detection of *Tg*AAP with fluorescently labeled anti-*Tg*AAP mouse polyclonal antibodies indicated that *Tg*AAP localized in the cytoplasm of the *T. gondii* wild-type RH strain, but was not expressed in the Δ*TgAAP* strain (Fig. 3).

### Loss of *TgAAP* affects attachment/invasion and growth *in vitro*

**Figure 4:**
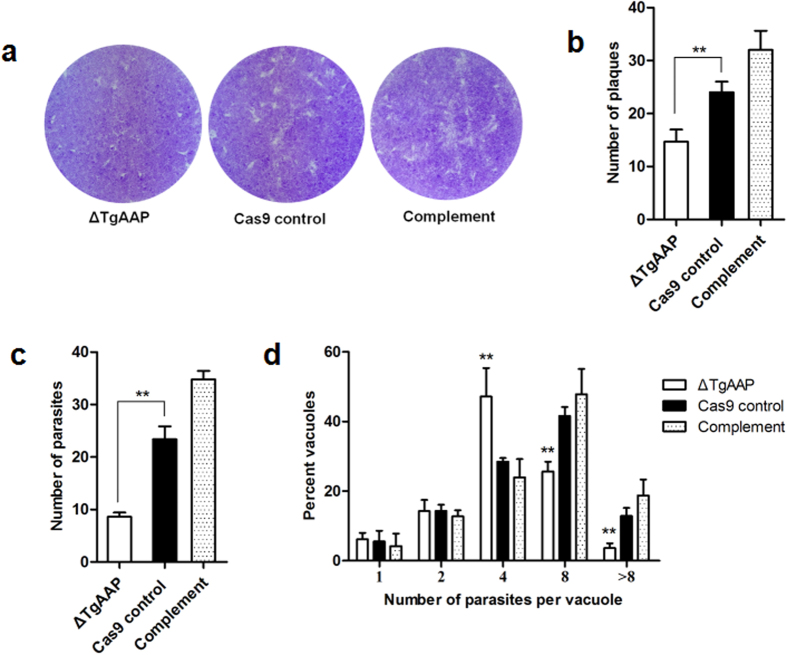
*Tg*AAP affects parasite invasion and growth *in vitro*. (**a**) Plaque assay examining the growth of parasites in HFFs. Plaques are visible as clear zones on a crystal violet-stained HFF monolayer background. (**b**) Quantification of Δ*TgAAP*, Cas9 control, and complement plaques using the digital images. (**c**) Invasion assay. HFF monolayers were inoculated (2 h) with Cas9 control, Δ*TgAAP*, or complemented parasites, and the mean number of parasites per field (y-axis) was counted after washing off the unattached parasites. At least 60 fields were scored for each strain. (**d**) Replication assay. Cas9 control, Δ*TgAAP*, or complemented parasite strains were grown for 24 h, and then the number of parasites per vacuole (x-axis) was counted. At least 100 vacuoles were scored for each strain. The differences between samples were evaluated by Student’s t-test (n = 3). *P < 0.05, **P < 0.01.

Aminopeptidases are exopeptidases that catalyze sequential removal of amino acids from the N-termini of peptides. These enzymes play major roles in regulating the balance between catabolism and anabolism in all living cells^20^. Since aminopeptidases can affect cellular metabolism, we anticipated that *Tg*AAP plays a role in parasite growth. To assess this, we tested the ability of *TgAAP*-knockout strains to form plaques on human foreskin fibroblast (HFF) monolayers. The Δ*TgAAP* strain was able to produce plaques (Fig. 4a), suggesting that the *TgAAP* gene is not essential for growth. During repeat experiments, plaques produced by the Δ*TgAAP* strain were always smaller and less numerous (Fig. 4a,b) than those produced by the Cas9 control strain (Student’s t-test, *P* < 0.05, n = 3). These results suggested that the parasite attachment/invasion and replication were reduced after *TgAAP* deletion. To further investigate whether the complemented strain would recover the ability to invade, intracellular parasite numbers were scored by microscopic observation (Fig. 4c). The mean number of parasites per field of view was significantly lower in the Δ*TgAAP* strain when compared with the Cas9 control strain 2 h post infection (Student’s t-test, *P* < 0.01, n = 3). These phenomena were reversed in the complemented strain. We also performed growth assays and scored the number of parasites per vacuole (Fig. 4d). Growth assays showed that a significant percentage of vacuoles in the Δ*TgAAP*-infected group contained tachyzoites that exhibited a reduced ability to divide 24 h post infection. Unlike the control and complemented strains, significantly more vacuoles containing four tachyzoites were seen in Δ*TgAAP* compared with the Cas9 control strain (Student’s t-test, *P* < 0.01, n = 3), with a significantly reduced number of eight-tachyzoite vacuoles (Student’s t-test, *P* < 0.01, n = 3). More parasitophorous vacuoles (PVs) containing more than eight tachyzoites were detected in the Cas9 control and complemented parasites. By contrast, fewer PVs containing more than eight tachyzoites were detected in the cells inoculated with Δ*TgAAP* parasites (Student’s t-test, *P* < 0.01, n = 3). Together, these results indicate that *TgAAP* plays an important role in parasite attachment/invasion and growth.

### Specific enzyme activity in Δ*TgAAP*

**Figure 5:**
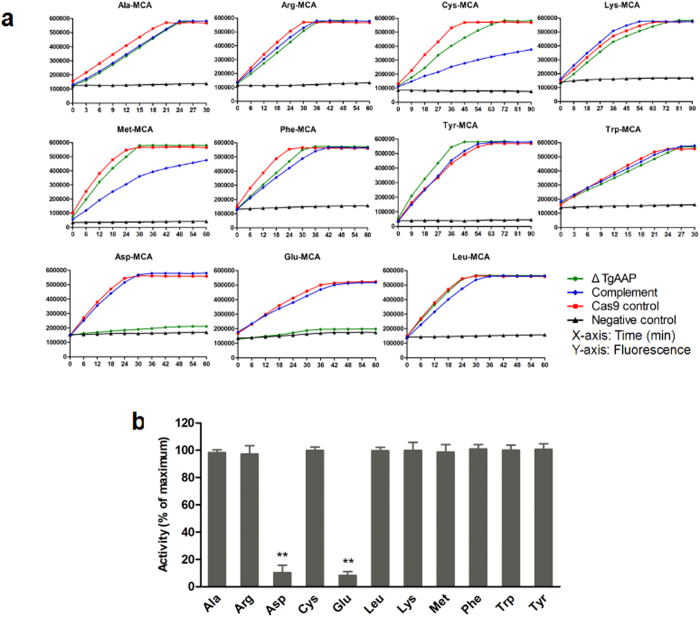
Enzyme activity in knockout and complemented parasite strains. (**a**) Cellular aminopeptidase activity with different fluoropeptide substrates, including AAP-specific substrates H-Asp-MCA and H-Glu-MCA. Enzyme activity curves of whole protein lysates from various parasite strains are shown and were generated using GraphPad Prism v. 5.0c (GraphPad Software, San Diego, USA). Enzyme activities were recorded at 3 min intervals until maximum activity levels were reached. Samples devoid of protein lysates were used as negative controls. All experiments were independently repeated three times (each in triplicate), and representative curves are shown. (**b**) Activities in *TgAAP*-knockout parasites toward different substrates (x-axis) calculated as percentages of the activity in Cas9 control lysates (y-axis). Student’s t-test was used for calculation of significance between groups. *P < 0.05, **P < 0.01 (n = 3).

Previous studies demonstrated that AAPs have a strict preference for the N-terminal acidic amino acids Glu and Asp^12^. We performed substrate-cleaving assays to investigate this specific enzymatic activity in the *TgAAP* deletion strain. Specific fluorescent substrates H-Asp-MCA and H-Glu-MCA were used. H-Leu-MCA and eight other substrates were used to detect the activities of other enzymes in Δ*TgAAP*, Cas9 control, and complemented strains. The results indicated that *TgAAP* deletion led to a significant decrease in AAP-specific enzymatic activity compared with the Cas9 control (*V*_max_; Student’s t-test, *P* < 0.01, n = 3) but the cleavage of H-Glu-MCA and H-Asp-MCA in the complemented strain was restored to Cas9 control levels (Fig. 5a). Exopeptidase activities and reaction rates with respect to Ala-, Arg-, Cys-, Lys-, Met-, Phe-, Tyr-, Trp-, and Leu-MCA were higher in Δ*TgAAP*, Cas9 control, and complemented strains. To further examine whether *TgAAP* knockout impacted other enzyme activities in *T. gondii* RH, enzyme activity in Δ*TgAAP* was assessed using 11 substrates (Fig. 5a) and compared with the Cas9 control (Fig. 5b). Except for a decreased activity toward H-Glu- and H-Asp-MCA (8.33% and 10.37% of the Cas9 control, respectively), no significant difference was observed between the two strains with respect to enzymatic activities toward other substrates, thus indicating the substrate specificity of *Tg*AAP.

### Inhibitor assay

**Figure 6:**
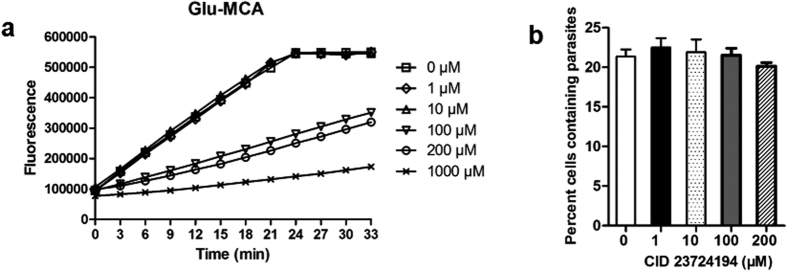
Susceptibility of r*Tg*AAP and *T. gondii* to CID 23724194. (**a**) r*Tg*AAP inhibition by CID 23724194 (Vitas-M Laboratory). The inhibition was studied at final drug concentrations of 0, 1, 10, 100, and 1000 μM, in reaction mixtures containing AAP substrate H-Glu-MCA. Data points indicate mean activity ± SD (n = 3). (**b**) Parasite growth inhibition as assayed by FACS. *Toxoplasm*a-infected host cells were quantified after 3 days of incubation with final CID 23724194 concentrations of 0, 1, 10, 100, and 200 μM. The experiments were repeated independently three times, each repeated in triplicate. Representative curves are shown in (**a**).

*In vitro* inhibition assay of r*Tg*AAP using CID 23724194, identified as a *Pf*M18AAP inhibitor^21^, is shown in Fig. 6a. r*Tg*AAP activity was partly inhibited by 100 μM CID 23724194. The activity of r*Tg*AAP was inhibited by more than 50% in the presence of 1 mM drug. The effect of drug concentration on parasite growth is shown in Fig. 6b. As the concentration of the drug was increased, no change in the percentage of *Toxoplasma*-infected host cells was observed (Student’s t-test, *P* > 0.05, n = 3). This indicated that CID 23724194 inhibited r*Tg*AAP, but this did not affect the growth of the parasite.

## Discussion

Similarly to *Pf*M18AAP, *Tg*AAP is the only AAP encoded in the genome of *T. gondii*. A recent study proved that *T. gondii* ingests and digests host cytosolic proteins. Disruption of this process attenuates the virulence of the parasites^22^. In addition, deletion of *Tg*LAP severely affects the growth of the parasites. Taken together, these phenomena suggest the importance of protein digestion by these enzymes in parasite development. This study reports the characterization of *Tg*AAP. The amino acid sequence of *Tg*AAP was predicted to contain conserved Zn-binding sites and substrate-binding/catalytic sites. The structure of *Pf*M18AAP revealed a dodecameric assembly that forms a tetrahedron shape^23^. The dodecameric enzyme is formed by interactions of four *Pf*M18AAP trimers with an internal M18 family active site cavity comprising four trimer cones, each with three active sites^19^. To assess the multimeric nature of *Tg*AAP, r*Tg*AAP was analyzed by native PAGE, which revealed that our recombinant enzyme exists in solution mainly as a dodecamer, partially in a trimeric and monomeric form. The multimeric nature of TgAAP is similar to that of *Pf*M18AAP. We also predicted a 3D model of *Tg*AAP that exhibited the highest homology with the crystal structure of human AAP (DNPEP, template: 4dyo.1.A, sequence identity: 44.9%)^24^ using the auto-model method (Fig. 3S). It is risky to develop new drugs targeting this enzyme. However, it is possible to identify drugs that target the non-conserved part of this enzyme. *Tg*AAP trimers and monomer structures were predicted separately from the tetrahedron by comparison with the X-ray crystal structure of a *Pf*M18AAP monomer. Structural prediction of the *Tg*AAP model suggested that the protein is a canonical member of the M18 aminopeptidase family. *Pf*M18AAP is expressed in the cytosol and is also trafficked out of the parasite into the surrounding PVs. It has therefore been speculated that *Pf*M18AAP plays a role in processing proteins or peptides in transit from the PV to the parasite cytosol^12^. We found that *Tg*AAP is also expressed in the cytosol. We previously characterized another toxoplasma aminopeptidase, M17 leucine aminopeptidase *Tg*LAP^25^, an exopeptidase with specificity toward N-terminal hydrophobic residues Leu and Phe that also localizes to the PVs. These two enzymes function in concert in protein catabolism in mammalian cells^15^. The free amino acids released during the process are likely used in anabolism and facilitate protein synthesis in the rapidly growing intracellular parasite.

Previous biochemical assays of the malarial AAP, *Pf*M18AAP, demonstrated that the enzyme requires metal ions for its activity and has a strict preference for N-terminal acidic amino acids Glu and Asp^12^. To investigate substrate specificity of *Tg*AAP, we performed H-Glu-/Asp-MCA substrate activity assays using recombinant *Tg*AAP expressed in *E. coli*. The results of an assay testing the effect of metal ions on enzyme activity indicated that Co^2+^ was the preferred metal cofactor for r*Tg*AAP, followed by Mn^2+^. This is different from the reported activity of *Pf*M18AAP^12^, enhanced only by Co^2+^. The substrate specificity assay indicated that r*Tg*AAP had a strict preference for H-Asp-MCA and H-Glu-MCA, although the catalytic efficiency of the enzyme was higher with H-Glu-MCA than with H-Asp-MCA. This was consistent with the finding that the initial reaction rate with H-Glu-MCA was higher than with H-Asp-MCA.

In *P. falciparum*, antisense knockdown experiments had previously identified a lethal phenotype associated with *Pf*M18AAP, suggesting that the enzyme is a valid target for new antimalarial therapies^26^. Another study reported that this enzyme is not dispensable for parasite growth through the disruption of this gene in the genome of *Plasmodium*^18^. To assess whether *Tg*AAP may be a candidate drug target for parasite growth control, we constructed a *TgAAP*-knockout strain and analyzed its phenotype. The absence of *TgAAP* hindered parasite invasion and growth but was not lethal to *T. gondii*. Therefore, we conclude that either the low activity of other aminopeptidases is able to sustain slower growth of the parasites or the AAP activity is not essential for parasite growth. High throughput screening was used to identify potent and selective inhibitors of AAPs. Compound CID 23724194 showed anti-parasite potency in malaria growth assays^11^. CID 23724194 efficiently inhibited *Pf*M18AAP activity in parasite lysates (96% at 10 μM) and efficiently inhibited parasite growth (IC50 = 1.3 μM). However, this inhibitor is almost completely ineffective against *Tg*AAP (more than 50% Glu-MCA cleavage inhibition at 1 mM). This may be due to differences in the protein sequence or spatial structure between *Pf*M18AAP and *Tg*AAP. The parasite growth inhibition assay revealed no significant difference between the percentages of *Toxoplasma*-infected host cells with increasing drug concentrations. In general, CID 23724194 is not a lead compound. Therefore, our results suggest that *Tg*AAP may only be used as an adjuvant drug target. Previously, we reported the function of LAP by deleting *Tg*LAP using the CRISPR/Cas9 genome editing technique. Our results indicated that *Tg*LAP was not essential, but played important roles in growth, invasion, and virulence. Accordingly, from the perspective of new anti-parasite drug development, M18AAP, along with M1 and M17, remains a protein of high interest^27^,^28^. Until now, we have only tested two metal aminopeptidases, *Tg*LAP and *Tg*AAP, and neither was essential to the parasite. Considering the importance of aminopeptidases for parasite growth and invasion, we believe that *Tg*LAP and *Tg*AAP, together or with other aminopeptidases, may serve as ideal targets. Recently, an “open collaboration” has been pursued in search of new drugs to manage neglected tropical diseases. Such initiatives advance the screening of additional molecular targets in *Plasmodium*, including *Pf*M18AAP, *Pf*M17LAP, and *Pf*M1^28^. These studies confirmed the feasibility of simultaneous targeting of two or more drug targets. It is yet to be determined whether aminopeptidases are suitable candidates for such drug development strategies for toxoplasmosis treatment.

It has been reported that AAPs of the M18 family are required for the removal of N-terminal acidic amino acid residues, Glu and Asp, that cannot be removed by M17 LAP or other aminopeptidases^29^,^30^. Similar conclusions may be drawn from our study: the substrate activity (H-Glu- and H-Asp-MCA) was almost completely lost in the Δ*TgAAP* strain. This also indicated that no other enzyme complemented the *Tg*AAP activity. These studies revealed the presence of additional aminopeptidases that could remove amino acids such as Ala, Arg, and Lys, not cleaved by *Tg*AAP or *Tg*LAP. Other aminopeptidases encoded in the malaria genome, for example, M1 membrane aminopeptidase N^30^, can cleave off these amino acids (Ala, Arg, and Lys), and thus complete the line-up of exopeptidases required for the total degradation of proteins to free amino acids in the parasite cytosol.

To investigate the essentiality of *Tg*AAP in *T. gondii*, we created a knockout/transgenic parasite line using the new DNA editing technology, the CRISPR/Cas9 system^31^,^32^,^33^. *TgAAP* deletion was successful, as evidenced by the absence of a corresponding specific protein band in the western blotting experiment and significantly decreased AAP-specific enzyme activity when compared with Cas9 control parasites. The method was also highly specific because the relative activity of *Tg*LAP was unaffected in the *TgAAP* deletion strain. Phenotype assay revealed that *TgAAP* deletion inhibited cell growth, leading to smaller parasite plaques, and resulting in a reduced substrate cleavage rate. However, the phenotype of the *TgAAP* deletion mutant was not as pronounced as the phenotype of the corresponding *Pf*M18AAP knockdown mutant^12^. Whether knocking out *TgAAP* or *TgLAP* would impact the anabolism of critical invasive proteins involved in parasite invasion requires further investigation.

Aminopeptidases have been treated as tractable *Plasmodium* targets for antimalarial drug development^34^,^30^,^28^. The function of other *T. gondii* aminopeptidases should also be investigated in the future, to establish a foundation for designing multiple-target anti-toxoplasma drugs to explore malaria treatments.

## Materials

### Parasite strains and growth conditions

The *T. gondii* RH strain (with *HXGPRT* deleted by homologous recombination; the strain is a generous gift from Dr. X. Xuan) and parasite mutants used in this study were maintained by growth in Vero cells or HFFs cultured in Dulbecco’s Modified Eagle Medium (DMEM, Gibco, Invitrogen) supplemented with fetal bovine serum (FBS; 2%), penicillin/streptomycin (1%), gentamicin (10 μg/mL), and glutamine (10 mM; Thermo, Fisher Scientific, Waltham, MA), at 37 °C in an air/CO_2_ (5%) environment. To purify *T. gondii* tachyzoites, parasites and host cells were washed in cold phosphate-buffered saline (PBS), and the final pellets were suspended in cold PBS and passed three times through a 27-gauge needle. The parasites were then passed through 5.0 μm-pore filters (Millipore, USA), washed twice with PBS, and stored at −80 °C until use.

### Sequencing *T. gondii* RH TgAAP

The *TgAAP* full-length sequence was amplified from a *T. gondii* RH strain cDNA library. Primers *Tg*AAPFwd (*Bam*HI site underlined) (5′-CGCGGATCCATGCAGACTGGCACAGAACTC-3′) and *Tg*AAPRev (*Hin*dIII site underlined) (5′-CCCAAGCTTTTACATGCCCTTGTAGCTATTGTC-3′) were designed based on the predicted *TgAAP* sequence (ToxoDB accession number: TGGT1_297970), upstream from the predicted start site and downstream from the predicted stop codon, respectively. The *TgAAP* fragment was amplified using KOD High-Fidelity DNA polymerase (TOYOBO Shanghai Co., Ltd.), cloned into pGEM-T Easy vector (Promega, France), and sequenced.

### Recombinant *Tg*AAP and anti-*Tg*AAP polyclonal antibody production

*Tg*AAP cDNA was amplified using the following primers: Cold*Tg*AAPFwd, 5′-CTCGAGGGATCCGAATTCATGCAAATGCAGACTGGCAC-3′ (*Eco*RI site underlined); and pCold*Tg*AAPRev, 5′-GTCGACAAGCTTGAATTCTTACATGCCCTTGTAGCTAT-3′ (*Eco*RI site underlined). The PCR fragment fused with GST at the N-terminus was cloned into the pCold III vector (Takara Bio Inc., Dalian, China). The verified plasmids were transformed into *E.* coli BL21. The culture were induced by treatment with 1 mM IPTG at an OD_600_ of ~0.5 and cultivated at 25 °C for 20 h. After centrifugation, cells were resuspended in PBS and lysed by ultrasonic treatment. Purification of r*Tg*AAP was performed using glutathione resin (GenScript, Piscataway, USA), according to the manufacturer’s instructions. r*Tg*AAP fused with GST was eluted with a buffer containing reduced glutathione (20 mM; GE Healthcare, Piscataway, USA) and dialyzed against Tris-HCl (50 mM, pH 8.0). The purified r*Tg*AAP was resolved on SDS-PAGE and native PAGE (Novex^®^ System, Invitrogen), as previously described^35^. The concentration of purified r*Tg*AAP was measured with the BCA Protein Assay Kit (Thermo Scientific Pierce, USA). Mice were immunized three times at 2 week intervals with GST-tagged r*Tg*AAP peptide (100 μg per injection) formulated in Freund’s Complete and Incomplete Adjuvant^6^. Sera were collected 14 days after the last immunization.

### Enzyme activity and kinetics

Enzymatic activity of r*Tg*AAP was determined by measuring the rate of Glu or Asp release from fluorogenic substrates H-Glu-MCA or H-Asp-MCA (Bachem, Bubendorf, Switzerland), respectively. MCA release was measured using the EnSpire Multimode Plate Reader (PerkinElmer, Turku, Finland), at 355 and 460 nm for both emission and excitation. The experimental data were plotted using GraphPad Prism v. 5.0c (GraphPad Software, San Diego, USA). To determine enzyme sensitivity to metal ions, *Tg*AAP activity was investigated after pre-incubating the enzyme (0.3 μM) at 37 °C for 30 min in Tris-HCl (50 mM, pH 7.5) containing 1 mM specified Co^2+^ (Sigma-Aldrich, St Louis, USA).

*K*_*m*_ (Michaelis-Menten constant) and V_max_ (maximum velocity) values of r*Tg*AAP were determined by incubating the enzyme (0.3 μM) in the presence of increasing concentrations of various fluorogenic substrates (Bachem, Bubendorf, Switzerland) at 37 °C. The initial velocity was calculated from the slope of the linear range of fluorescence vs. time curve. The average *K*_*m*_ and V_max_ values were calculated with standard errors from three independent experiments.

### Construction of *T. gondii* transgenic knockout and complementing plasmids

The structure of the pSAG1::CAS9-U6::sgUPRT plasmid (Addgene, #54467) has been described previously^31^. In this vector, Cas9 protein fused with GFP is expressed under the control of a SAG1 promotor. We constructed a knockout vector targeting *TgAAP* by modifying this plasmid. The backbone sequence, except the single guide RNA (gRNA) cassette, was amplified from pSAG1::CAS9-U6::sgUPRT and self-ligated after digestion with *Pme*I. A *DHFR* cassette was then inserted into the *Kpn*I site, and the resultant plasmid was designated pCD. Finally, a gRNA cassette targeting *TgAAP* (gRNA sequence: 5′-AGAACGAGGATATCGTTGAG-3′) under the control of the *Tg*U6 promoter was cloned into the *Pme*I site. The final plasmid was designated pCD-*Tg*AAP.

Plasmid pBluescript II containing the *HXGPRT* expression cassette (derived from pHXNTPHA plasmid, a generous gift from Dr. X. Xuan) and a GFP cassette under the control of the GRA1 promotor (derived from PDMG^33^) was used to construct the complementing plasmid. To validate and identify the monoclonal complemented strain, the GFP expression cassette was inserted into a the plasmid. The plasmid was designated pBH. To construct the complementing plasmid, the synonymous codon *TgAAP* gene (syno*Tg*AAP, containing synonymous codons GTG and GAA in the target gRNA sequence adjacent to the PAM site) was amplified using primers syno*Tg*AAPFwd (5′-GAACGAGGATATCGTGGAATGGGACTTGTG-3′) and syno*Tg*AAPRev (5′-CACAAGTCCCATTCCACGATATCCTCGTTC-3′), and inserted into the *Pme*I site of the pBH vector using the ClonExpress™ II One Step Cloning Kit. In the constructed plasmid, syno*Tg*AAP expression was regulated by the *GRA1* promoter. The C-terminus of syno*Tg*AAP was fused to mCherry tag (synthesized by GeneScript Co., Ltd.) by hierarchical fusion PCR. The final plasmid was designated pBH-syno*Tg*AAP.

### Transient transformation and Cas9-mediated gene disruption and complementation

Transient transformation with the plasmid (pCD-TgAAP) was achieved by transfecting freshly lysed tachyzoites with circular DNA (10 μg)^36^, and immediate infection of Vero cells. Selection of the transfected parasites that acquired resistance to pyrimethamine (1 μM, in ethanol) was performed as described earlier^37^. For cloning purposes, the transformed parasites were serially diluted into a culture of Vero cells grown on 96-well plates without selection. Single-plaque wells were replica-passaged for propagation or for western blotting, and indirect immunofluorescence detection with anti-r*Tg*AAP antibodies was used to determine whether the *TgAAP* gene was disrupted. To generate Δ*TgAAP*/syno*Tg*AAP complemented parasites, 10^7^ Δ*TgAAP* parasites were transfected with pBH-syno*Tg*AAP plasmid (10 μg) and selected with mycophenolic acid (25 μg/mL) and xanthine (50 μg/mL). After three generations, parasites expressing syno*Tg*AAP were used in *in vitro* invasion and replication assays. We also constructed and screened a *T. gondii* RH strain that was transformed with plasmid pCD-HXGPRT (*HXGPRT* gRNA sequence: 5′-GTGGAGACCATTGGGTCACT-3′), named Cas9 control. The resultant strain was used as a control.

### Western blotting

Samples for western blotting were obtained by gentle centrifugation of extracellular parasites and incubating them with RIPA buffer (50 mM Tris-HCl, pH 8.0, 150 mM NaCl, 1% Triton X-100, 0.5% sodium deoxycholate, 0.1% SDS, and 1 mM EDTA) containing a protease inhibitor cocktail (Calbiochem, USA) for 20 min on ice, to lyse them. Next, the samples were centrifuged for 10 min at 15,000 × *g* at 4 °C, and Laemmli buffer was added to the supernatant. Unless indicated otherwise, an equivalent of 10^7^ parasites was loaded per SDS-polyacrylamide gel lane and immunoblotting was performed. Briefly, parasite extracts were resolved on a 10% SDS-polyacrylamide gel, transferred to a PVDF membrane, and probed with mouse anti-r*Tg*AAP serum (1:500) as a primary antibody. The mCherry protein was detected using an anti-mCherry mouse monoclonal antibody (1:5000) (Abbkine, California, USA) as the primary antibody. Then, a horseradish peroxidase-conjugated goat anti-mouse antibody (1:5000) (Jackson Immunoresearch, West Grove, Pennsylvania, USA) was used as the secondary antibody. Chemiluminescent signals were developed with the SuperSignal West Pico Chemiluminescent Substrate (Thermo Scientific, Waltham, USA).

### Immunofluorescence

Immunofluorescence assays were carried out as described previously^25^. Briefly, after 16–24 h of incubation, HFF monolayers infected with *T. gondii* wild-type RH or Δ*TgAAP* parasites were fixed with paraformaldehyde (4%) prepared in PBS for 15 min and then permeabilized with Triton X-100 (0.3%) prepared in PBS for 10 min. Cells were then incubated with mouse anti-r*Tg*AAP serum (1:200) as a primary antibody. An Alexa 488-conjugated goat anti-mouse fluorescent antibody (1:500) (Jackson ImmunoResearch) was used as the secondary antibody. The parasite nuclei were visualized with DAPI. Samples were examined with a confocal microscope.

### Attachment/invasion assay

To measure parasite attachment/invasion rates, freshly lysed Cas9 control, knockout, and complemented parasites were filtered and used to inoculate HFF cell monolayers in 6-well culture plates (Costar, USA; 10^6 ^parasites/well, three wells per each strain). Parasites were allowed to invade HFF cells for 2 h (37 °C, 5% CO_2_), and extracellular parasites were washed off with PBS. Then, mean numbers of parasites per field (60 fields/well) were counted under a microscope. Three independent experiments were performed, and each strain was assayed in triplicate within each experiment.

### Replication assay

To directly compare the growth of Cas9 control and knockout parasites, plaque assays were performed. Purified parasites were used to infect fresh HFF monolayers seeded in 6-well plates and grown for 7 days without shaking (100 parasites/well). Cells were then fixed with ethanol (70%) and stained with crystal violet (0.1%). Plaques were scanned using an Epson scanner and analyzed as previously reported^31^. Three independent experiments were performed, and each strain was assayed in triplicate within each experiment. To investigate replication rates of Δ*Tg*AAP, Cas9 control, and complemented parasite lines, freshly lysed tachyzoites were collected, filtered, and used to inoculate Vero cell monolayers in 6-well culture plates (10^6 ^parasites/well). Parasites were allowed to invade for 2 h under normal growth conditions (37 °C, 5% CO_2_), following which, extracellular parasites were washed away with PBS, and the parasites were incubated for another 24 h. The number of vacuoles containing the indicated number of parasites (i.e., 1, 2, 4, 8, or >8 cells) was counted in ≥100 vacuoles from three separate wells per experiment. Three independent experiments were conducted, and the results were combined and graphed.

### Native enzyme activity assay

To determine the native *Tg*AAP enzyme activity in the knockout and complemented lines, freshly lysed tachyzoites were purified and collected by centrifugation. Proteins were extracted using lysis buffer (20 mM Tris-HCl, pH 8.0, 137 mM NaCl, 1% Nonidet P-40, and 2 mM EDTA) and quantified using a BCA Protein Assay Kit (Pierce, Bonn, Germany). Parasite proteins (10 μg) were added to 200 μL of Tris-HCl buffer (50 mM, pH 7.5) before a specific substrate (0.1 mM) was added. Relative fluorescence levels were assessed every 3 min for a total of 60 min. To determine the effect of *TgAAP* deletion on the activity of other cellular aminopeptidases, 11 substrates were used. For each substrate, maximum enzyme activities of Δ*TgAAP* and Cas9 control strains were compared.

### Inhibition assay

To determine the inhibitory effect of CID 23724194 (Vitas-M Laboratory, Catalog # STK091533), r*Tg*AAP was pre-incubated with CID 23724194 for 1 h at 37 °C before the addition of the fluorogenic substrate H-Glu-MCA. Relative enzyme inhibition levels were assessed with various final CID 23724194 concentrations (1, 10, 100, 200, and 1000 μM). To determine the inhibitory effect of CID 23724194 on parasite growth, Cas9 control strain cultured in Vero cells was incubated with the compound at various final concentrations (1, 10, 100, and 200 μM) for 3 days. The percentages of *Toxoplasma*-infected host cells were then evaluated using FACS.

## Additional Information

**How to cite this article**: Zheng, J. *et al.* Characterization of aspartyl aminopeptidase from *Toxoplasma gondii.* *Sci. Rep.* **6**, 34448; doi: 10.1038/srep34448 (2016).

## Supplementary Material

Supplementary Information

## Figures and Tables

**Table 1:** Effect of divalent metal ions on rTgAAP activity.

Metal ion	Concentration (mM)	Activity (units/s)^a^
Co^2+^	1	377.76 ± 56.36
Mn^2+^	1	151.3 ± 26.44
Fe^2+^	1	133.45 ± 68.28
Ni^2+^	1	116.89 ± 39.34
Mg^2+^	1	62.47 ± 7.08
Ca^2+^	1	44.52 ± 9.12
Cu^2+^	1	3.78 ± 0.478
Zn^2+^	1	2.81 ± 0.516
Control (No metalion)	—	59.39 ± 2.72

^a^One unit was defined as picomoles of MCA released per milligram of recombinant protein. Data represent means ± SD from three independent experiments.

**Table 2:** Kinetic parameters for the hydrolysis of peptide substrates by rTgAAP.

Substrate	*k*_cat_ (×10^−2 ^s^−1^)^a^	*K*_m_ (μM)^a^	*k*_cat_/*K*_m_ (mM^−1 ^s^−1^)
H-Glu-MCA	139.67 ± 30.65	7.35 ± 0.73	190.16
H-Asp-MCA	109.99 ± 6.48	12.84 ± 0.21	85.66
H-Asn-MCA	48.35 ± 1.26	24.32 ± 0.26	19.86
H-His-MCA	20.84 ± 0.43	88.70 ± 2.53	2.35
H-Ser-MCA	12.35 ± 0.86	92.87 ± 3.38	1.33
H-Pro-MCA	27.59 ± 2.37	98.55 ± 1.96	0.28

^a^Data represent means ± SD from three independent experiments.
