# Supplementary material for: Characterization of aspartyl aminopeptidase from Toxoplasma gondii
Source: Sci Rep. 2016 Sep 28;6:34448. doi: 10.1038/srep34448 (PMC5039622; doi:10.1038/srep34448)
Supplement: Supplementary Information [file srep34448-s1.doc]

**Characterization of aspartyl aminopeptidase from *Toxoplasma gondii***

Jun Zheng^1^, Ziying Cheng^1^, Honglin Jia^1*^, and Yonghui Zheng^1^

^1^Harbin Veterinary Research Institute, CAAS-Michigan State University Joint Laboratory of Innate Immunity, State Key Laboratory of Veterinary Biotechnology, Chinese Academy of Agricultural Sciences, Maduan Street 427, Nangang District, Harbin 150001, P.R. China.

***Corresponding author**

Honglin Jia

Harbin Veterinary Research Institute, CAAS-Michigan State University Joint Laboratory of Innate Immunity, State Key Laboratory of Veterinary Biotechnology, Chinese Academy of Agricultural Sciences, Maduan Street 427, Nangang District, Harbin 150001, P.R. China

Tel: +8618946066070

Fax:

E-mail: jiahonglin@caas.cn

**
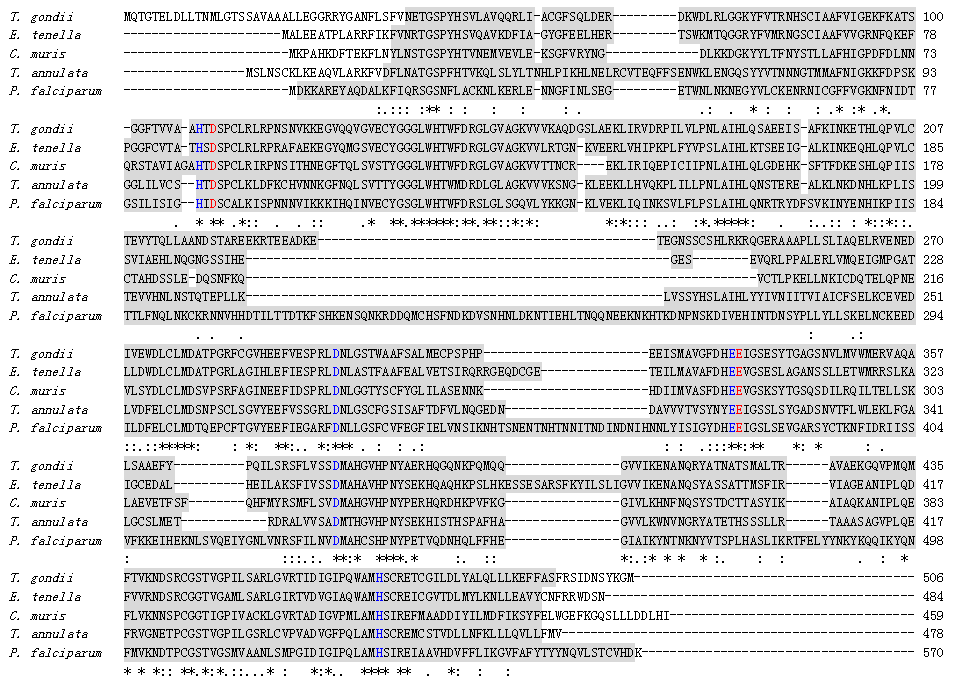
**

**(a)**

**
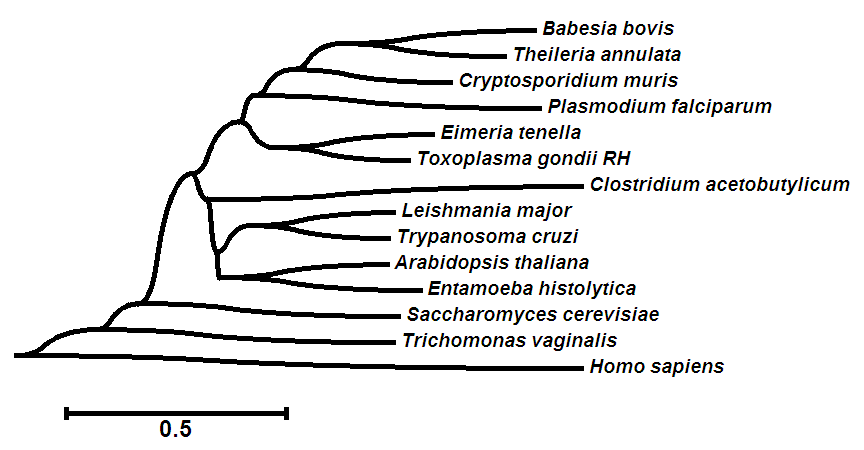
**

**(b)**

**Figure 1S.** **Alignment and phylogenetic tree of *Tg*AAP.** (**a**) Alignment of amino acid sequences of apicomplexan parasite AAPs. Sequences used in this study are as follows: *Toxoplasma gondii* RH AAP (identical to GT1 AAP, ToxoDB: TGGT1_297970), *Eimeria tenella* AAP (GeneDB: ETH_00026985), *Cryptosporidium muris* AAP (CryptoDB: CMU_010670), *Plasmodium falciparum* M18AAP (GeneDB: PF3D7_0932300), and *Theileria annulata* AAP (GeneDB: TA16610). Identical (*), 90% conserved (:), and 50% conserved (.) amino acids are indicated. Functional domains were predicted using the PFAM protein search algorithm in the SMART program (http://smart.embl-heidelberg.de/) and are highlighted in gray. *Tg*AAP conserved Zn-binding sites and substrate-binding/catalytic sites were predicted using BLAST MEROPS (http://merops.sanger.ac.uk/) and are marked in red and blue, respectively. (**b**) Phylogenetic tree of apicomplexan parasite AAPs and several other M18 family members. The scale at the bottom indicates the evolutionary distance between sequences. The tree was obtained using MEGA v. 6 program, Muscle alignment, and neighbor-joining method with bootstrap of 10,000 predicted sequences from the MEROPS peptidase database (http://merops.sanger.ac.uk/) and GeneDB (http://www.genedb.org/). *Leishmania major* strain *Friedlin* (GeneDB: LmjF.29.2360), *Trypanosoma cruzi* (GeneDB: TcCLB.508183.4), *Babesia bovis* (PiroplasmaDB: BBOV_IV011550), *Entamoeba histolytica* (AmoebaDB: EHI_106690), *Trichomonas vaginalis* (TrichDB: TVAG_392410), *P. falciparum Pf*M18AAP (MERNUM: MER024893), *Homo sapiens* ADAM18 (MERNUM: MER012230), *Clostridium acetobutylicum* ApeA protein (MERNUM: MER005460), (*Arabidopsis thaliana*)-like peptidase (MERNUM: MER003372), and *Saccharomyces cerevisiae* aminopeptidase I (MERNUM: MER001255) were used as outgroups.


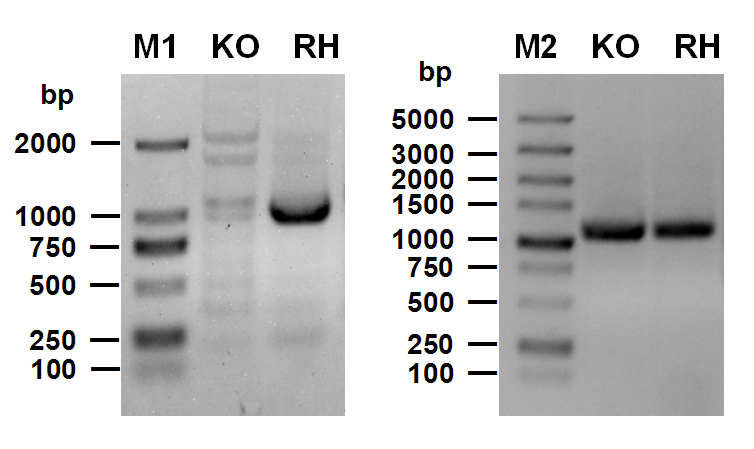


1. ***Tg*AAP**
2. ***Tg*LAP**

**Figure 2S. PCR analysis of *TgAAP*-knockout (Δ*TgAAP*) and wild-type *T. gondii* RH strain.** *TgAAP* was detected by PCR in parasite genomic DNA using specific primers. PCRs had a total volume of 25 µl and contained 50 ng of parasite genomic DNA, 0.2 μM primers, 0.2 mM of each dNTP, 2 M MgCl_2_, 0.5 U of ExTaq polymerase (TaKaRa), and 1× PCR buffer. Thermal cycling included a denaturation step of 5 min at 95°C, 30 cycles of 30 s at 95°C, 30 s at 58°C, and 1 min at 72°C, and a final extension step at 72°C for 10 min. PCR products were analyzed by electrophoresis on 1% agarose gels and visualized by ethidium bromide staining. PCR product containing gRNA target sequence was 1000 bp long. A specific amplified band was observed in RH wild-type parasites (left image, lane RH) but not in ΔTg*AAP* strain (left image, lane KO). Many bands were seen in the KO lane. Four bands were more prominent (red arrows). After purifying, sequencing, and BLAST analysis in ToxoDB, they were all identified as non-specific amplification products. These results verified deletion of the targeted gene. PCR was performed using specific primers AAPF (5′-CGAACAGAGGAGGCAGACAAGG-3′) and AAPR (5′-CAAGCGAGGCGACTCAACGAACT-3′). *TgLAP*-specific primers (LAPF, 5′-GGTGGAGAAACTTACGCTTTTCACGG-3′; and LAPR, 5′-CGAAGCAGATGCCCTTGCCCACAAAC-3′) were used for amplification control (right image). M1: 2000 DNA marker (TaKaRa). M2: 5000 DNA marker (TaKaRa). KO: Δ*TgAAP* strain. RH: wild-type RH strain.

**Figure 3S.** **Analysis of the presumed *Tg*AAP model and comparison with *Pf*M18AAP.** To predict the protein spatial structure, the amino acid sequence (ToxoDB: TGGT1_297970) of *Tg*AAP was used to generate a homology-based molecular model using the SWISS MODEL protocol (http://swissmodel.expasy.org/). We used the experimentally characterized human aminopeptidase as a model to predict the structure of *Tg*AAP. The 3D models of *Tg*AAP were viewed and analyzed using PyMOL (version 0.99rc6), and the models were compared using Swiss-PdbViewer (version 4.10) (Guex et al., 2009). The domains and metal ion-binding sites were predicted by the NCBI BLAST tool. The tetrahedron structure (left) of *Tg*AAP was derived using the SWISS-MODEL program. The trimer (central image) and monomer (shown in green in the image on the right) of *Tg*AAP were presumed and separated from the tetrahedron by comparison with the monomer X-ray crystal structure (shown in aquamarine in the image on the right) of *P. falciparum* M18AAP (PDB accession number: 4EME). Active sites in the *Tg*AAP model monomer are indicated by red, yellow, and blue in the trimer. Structural prediction of the *Tg*AAP model revealed that it was a canonical member of the M18 aminopeptidase family and exhibited a higher homology with the crystal structure of human aspartyl aminopeptidase (DNPEP, templata: 4dyo.1.A, sequence identity: 44.9%) (Chaikuad et al., 2012) generated using the SWISS MODEL protocol. Like human and *P. falciparum* M18AAP, *Tg*AAP was predicted to form a complex homododecameric tetrahedron quaternary structure (Fig. 1C, left), but exhibited subtle differences in the monomer structure compared with *Pf*M18AAP (Fig. 1C right). In addition, we also separated a trimer unit from the predicted molecular model of *Tg*AAP (Fig. 1C, central image). The trimer completely enclosed the three active sites (red, yellow, and blue) within a central cavity.
